# Effects of Sub-threshold Transcutaneous Auricular Vagus Nerve Stimulation on Cingulate Cortex and Insula Resting-state Functional Connectivity

**DOI:** 10.3389/fnhum.2022.862443

**Published:** 2022-04-14

**Authors:** Yixiang Mao, Conan Chen, Maryam Falahpour, Kelly H. MacNiven, Gary Heit, Vivek Sharma, Konstantinos Alataris, Thomas T. Liu

**Affiliations:** ^1^Center for Functional MRI, University of California San Diego, La Jolla, CA, United States; ^2^Department of Psychology, Stanford University, Stanford, CA, United States; ^3^Nēsos Corporation, Redwood City, CA, United States; ^4^Department of Neurosurgery, Hue University of Medicine and Pharmacy, Hue, Vietnam; ^5^Departments of Radiology, Psychiatry, and Bioengineering, University of California San Diego, La Jolla, CA, United States

**Keywords:** taVNS, resting-state functional connectivity, resting-state fMRI, cingulate cortex, insula

## Abstract

Transcutaneous auricular vagus nerve stimulation (taVNS), a non-invasive alternative to vagus nerve stimulation (VNS) with implantable devices, has shown promise in treating disorders such as depression, migraine, and insomnia. Studies of these disorders with resting-state functional magnetic resonance imaging (MRI) (rsfMRI) have found sustained changes in resting-state functional connectivity (rsFC) in patients treated with low frequency (1–20 Hz) taVNS. A recent study has reported reductions in pain scores in patients with rheumatoid arthritis after a 12-week treatment of high-frequency (20 kHz) sub-threshold taVNS. However, no studies to date have examined the effects of high-frequency sub-threshold taVNS on rsFC. The objective of this study was to determine whether high-frequency sub-threshold taVNS induces changes in rsFC using seed regions from the cingulate cortex and insula, brain regions that play a key role in interoception and processing of pain. With a single-blind placebo-controlled repeated measures experimental design, rsfMRI scans were acquired before and after 15 min of either sub-threshold taVNS treatment or a sham control. Significant taVNS-related changes in functional connections to the cingulate cortex were detected between the anterior cingulate cortex and right superior temporal gyrus and between the midcingulate cortex and right inferior parietal lobule. In addition, significant changes in functional connections to the insula were detected between the posterior insula and right precuneus and between the anterior insula and right cuneus gyrus. These results suggest that high-frequency sub-threshold taVNS can lead to sustained effects on the rsFC of brain regions involved in interoception and processing of pain in a cohort of healthy subjects. This study lays the foundation for future rsfMRI studies of high-frequency sub-threshold taVNS in clinical populations.

## 1. Introduction

Vagus nerve stimulation (VNS) with implantable devices is currently used as a treatment for disorders such as drug-resistant epilepsy and treatment-resistant depression. However, the cost and complications involved in implanting the devices have hindered the extension of VNS to a broader range of applications (Kraus et al., 2007; Dietrich et al., 2008; Fang et al., 2016; Badran et al., 2018). Thus, there is growing interest in non-invasive approaches, such as transcutaneous auricular vagus nerve stimulation (taVNS) (Fang et al., 2016; Rong et al., 2016; Garcia et al., 2017; Addorisio et al., 2019). In taVNS, an electrode on the surface of the ear delivers a small electrical current across the skin to the auricular branch of the vagus nerve. Stimulation of the auricular vagus nerve projects to nuclei in the brainstem and further activates additional brain areas through connected pathways, including regions involved in the regulation of the autonomic nervous system (Peuker and Filler, 2002; Kaniusas et al., 2019). Prior studies suggest that taVNS may have therapeutic benefits in the context of depression (Rong et al., 2016; Kong et al., 2018; Liu et al., 2020), migraine (Straube et al., 2015), and epilepsy (Stefan et al., 2012; Rong et al., 2014). Moreover, a recent open-label study of subjects with rheumatoid arthritis (RA) demonstrated that a 12-week treatment of high-frequency (20 kHz) sub-threshold taVNS was well-tolerated and led to clinically relevant changes in both disease biomarkers and symptoms (Marsal et al., 2021), indicating the potential of high-frequency (20 kHz) sub-threshold taVNS as a non-pharmacological alternative for the treatment of rheumatoid arthritis.

While the mechanisms through which taVNS causes changes to the brain are not completely understood, there has been growing interest in the use of blood oxygen-level dependent (BOLD) functional magnetic resonance imaging (MRI) (fMRI) to characterize the acute functional brain response to taVNS as well as sustained changes in functional brain connectivity. Task-based fMRI studies have shown significant taVNS-related changes in a wide range of brain regions that are involved in the afferent pathways of the vagus nerve, including the cingulate cortex, insula, hippocampus, amygdala, thalamus, cerebellum, nucleus tractus solitarius, and locus coeruleus (Kraus et al., 2007, 2013; Dietrich et al., 2008; Frangos et al., 2015; Yakunina et al., 2017; Badran et al., 2018; Zhang et al., 2019). As a complementary approach, resting-state fMRI (rsfMRI) studies have been used to characterize taVNS induced changes in resting-state functional connectivity (rsFC), typically calculated as the temporal correlation between the BOLD signals from different brain regions (Heuvel and Pol, 2010). To date these studies have been focused on cohorts of patients with clinical disorders, such as major depressive disorder (Fang et al., 2016; Liu et al., 2016), migraine (Zhang et al., 2019, 2020), and primary insomnia (Zhao et al., 2020; Zhang et al., 2021). Although the methods and results vary widely across the studies, the prior findings suggest that taVNS can lead to sustained changes in connectivity within networks that include the cingulate cortex, precuneus, amygdala, prefrontal cortex, locus coeruleus, thalamus, and hippocampus.

In this study of healthy subjects, we examined rsFC changes induced by 15 min of high-frequency (20 kHz) taVNS with duration and stimulation parameters similar to those of the daily dose employed in Marsal et al. (2021). In contrast to prior studies where the perceptible sensations associated with taVNS may have complicated the interpretation of the results, we applied taVNS at a sub-threshold level, such that subjects could not readily determine if they were receiving the treatment or a placebo. This approach removed somatosensory confounds and facilitated the use of a single-blind placebo-controlled design. Motivated by the taVNS-related reduction in pain scores reported by Marsal et al. (2021), we assessed treatment-related changes in rsFC using seed regions from the cingulate cortex and insula, brain regions that play a key role in interoception and processing of pain (Vogt, 2005; Craig, 2009; Wager et al., 2013).

## 2. Materials and Methods

### 2.1. Overview

**Figure 1:**
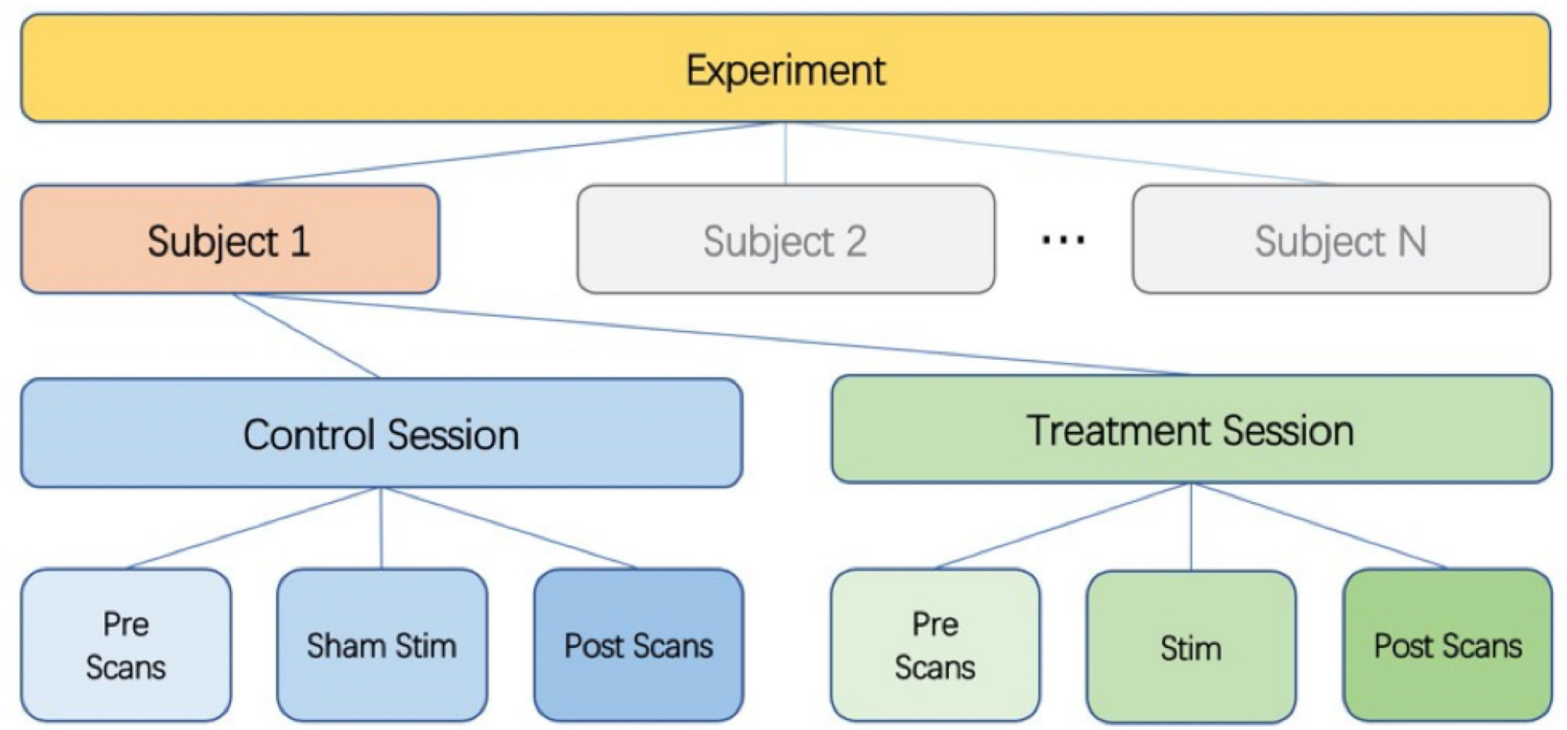
Diagram of experimental design. Each subject undergoes a control session and a treatment session. Each session consists of pre scans, a stimulation (sham for control session) section, and post scans.

We used a single-blind placebo-controlled experimental design. Twenty-seven healthy subjects were enrolled in this study after providing informed consent (14 females and 13 males, aged 19–40 years). The study was approved by the UCSD Institutional Review Board (IRB, project #190011), and informed consent was obtained from all participants. Figure 1 shows the overall experiment structure. Each subject participated in a control session and a treatment session, with the order randomized across subjects. The sessions were separated by at least 1 day to minimize taVNS carryover effects (Badran et al., 2018).

All scanning visits consisted of a pre scan section, a stimulation (or sham) section, and a post scan section. For the first six subjects, the subjects wore the stimulation earpieces throughout the entire session, and the stimulation section took place while they remained lying inside the scanner. To improve the subject experience, the protocol was slightly modified for the subsequent twenty-one subjects. These twenty-one subjects wore regular earplugs during the MRI scans, came out of the scanner for the stimulation (or sham) section, and then returned to the scanner with regular earplugs to finish the post scan section.

### 2.2. taVNS

Following the reporting standards recommended in Farmer et al. (2021), we report details of the taVNS implementation here. The taVNS stimulus was delivered using custom-fit ear pieces designed by Nēsos® (Redwood City, CA). A bipolar configuration was used and two hydrogel electrodes were placed on the ear piece surface interfacing with the cymba concha. The electrodes have identical circular shapes with radii of 0.3 cm, resulting in a contact area of 0.28 cm^2^ for each electrode. Stimulation was applied to the left ear, targeting vagal afferents in the cymba concha based on known vagus nerve anatomy (Berthoud and Neuhuber, 2000; Elenkov et al., 2000; Peuker and Filler, 2002). For all visits, the stimulation (or sham) section took place between the pre and post scan sections.

For both the control and treatment sessions, a sensation test was performed before the stimulation (or sham) section to determine a subject's threshold for perceiving the stimulation. The process for determining the threshold was the same one used in the prior study of Marsal et al. (2021), which entailed starting with a stimulus current of 1.0 mA and increasing by increments of 0.5 mA (up to a maximum of 5.0 mA) until the subject reported feeling a sensation. The stimulus current was then decreased by decrements of 0.5 mA until the sensation went away and then further decreased by 1 mA before retesting the threshold. Criteria for perceiving the stimulation include sensations of stinging, pricking, or warmth. This procedure was repeated until the subject reported feeling the stimulation at the same level three times. The sensation test was followed by a 15-min stimulation period. The stimulation was delivered continuously at 20 kHz, using biphasic square waves with pulse width of 20 μs. The stimulation amplitude was set at 75% of a subject's perceptual threshold (1.5–3.8 mA; impedance 3–9.5 kΩ) on the “active” stimulation day and was set to zero on the control day (“sham” condition). The order of “active” and “sham” stimulation days was randomized across subjects. The taVNS equipment is designed to be safe for human use and delivers charge-balanced, current-controlled stimulation. Safety of the 20 kHz stimulus has been demonstrated in a clinical trial (Marsal et al., 2021). Furthermore, safety in this study was assessed by recording any adverse events or discomfort reported by the subjects.

Since the stimulation was delivered at 75% of perceptual threshold, subjects were blinded to whether they were undergoing the sham or stimulus treatment. At the end of their second session, all subjects were asked whether they could identify their control and treatment sessions across their two visits.

### 2.3. MR Scans

Scans were acquired on a GE MR750 Signa Discovery 3.0 T system. Pre and post scan sections consisted of (1) a high-resolution anatomical scan (MPRAGE, flip angle = 8°, resolution = 1 mm^3^, FOV = 25.6 cm, TE = 2.92 ms, TR = 2,500 ms, matrix size = 256 × 256 × 208), (2) 6-min resting-state scans (flip angle = 52°, slice thickness = 2.4 mm, FOV = 21.6 cm, TE = 32 ms, TR = 960 ms, matrix size = 90 × 90 × 72, multiband acceleration = 6), and (3) arterial spin labeling scans. Only the anatomical and resting-state scans are analyzed here. The arterial spin labeling (ASL) scans are analyzed in a separate article (Chen et al., 2021). During all resting-state scans, subjects rested with their eyes open and gently focused on a fixation point.

### 2.4. MR Preprocessing

AFNI and FSL were used for MRI data pre-processing (Cox, 1996; Smith et al., 2004; Woolrich et al., 2009). The high resolution anatomical data were skull stripped and segmentation was applied to estimate white matter (WM), gray matter (GM) and cerebral spinal fluid (CSF) partial volume fractions.

The first 12 volumes (11.52 s) of the BOLD data were used as reference images for the multiband reconstruction and were discarded. Each functional scan went through: (1) susceptibility distortion correction and volume registration, (2) slice-timing correction, (3) spatial normalization to standard space (ICBM 2009a Non-linear Asymmetric template (Fonov et al., 2011), voxel size = 2 ×2×2 mm^3^), (4) spatial smoothing using a 4 mm FWHM Gaussian kernel, and (5) removal of nuisance regressors through linear regression. These nuisance regressors included: (1) linear and quadratic trends, (2) six motion parameters estimated during volume registration and their first derivatives, (3) RVHRCOR physiological (Chang and Glover, 2009; Chang et al., 2009) noise terms calculated from the cardiac and respiratory signals, and (4) five aCompCor regressors estimated from the union of the WM and CSF masks (Behzadi et al., 2007). The respiratory variation (RV) and heart rate (HR) correction (RVHRCOR) physiological noise terms were used to correct the BOLD signal for the effects related to low-frequency respiratory variation and heart rate changes (Chang and Glover, 2009; Chang et al., 2009). After regressing out the nuisance regressors, each voxel's signal was converted to a percent change signal and band-pass filtered (0.01–0.1 Hz) using a zero delay, fourth-order Butterworth filter.

To further reduce the confounding effects of head motion, motion censoring was performed. Framewise displacement (FD) was calculated based on six motion parameters estimated during volume registration as in Power et al. (2012), with lowpass filtering of the motion parameters (0.1 Hz) to correct for respiratory artifacts (Fair et al., 2020). Volumes with filtered FD larger than 0.1 mm were censored from rsFC analysis. To ensure that there is adequate data for rsFC analysis [a minimum of 5 min of data, as suggested in Dijk et al. (2010)], subjects were excluded if any one of their four runs had less than 313 volumes (300.48 s) after motion censoring.

### 2.5. Seed-based Full Brain Connectivity Analysis

**Figure 2:**
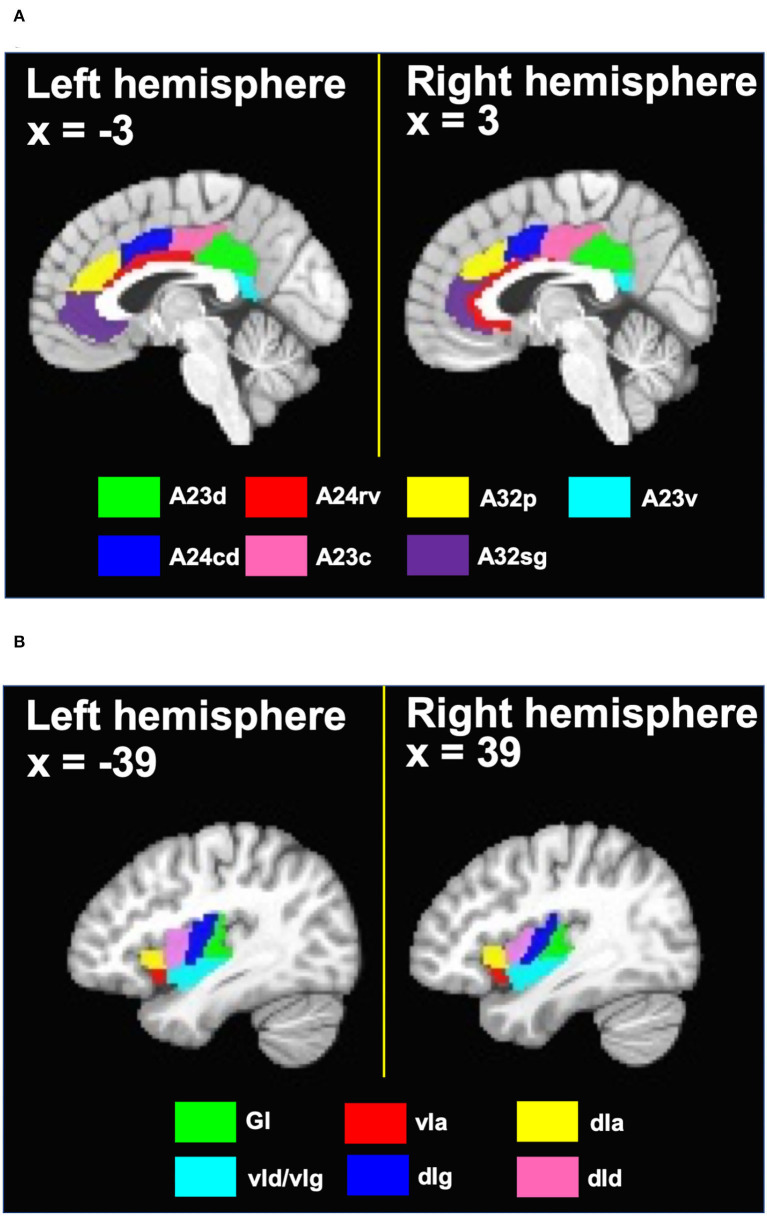
**(A)** Cingulate ROIs and **(B)** insula ROIs defined based on the Brainnetome atlas. A23d, dorsal area 23; A24rv, rostroventral area 24; A32p, pregenual area 32; A23v, ventral area 23; A24cd, caudodorsal area 24; A23c, caudal area 23; A32sg, subgenual area 32; GI, hypergranular insula; vIa, ventral agranular insula; dIa, dorsal agranular insula; vId/vIg, ventral dysgranular and granular insula; dIg, dorsal granular insula; dId, dorsal dysgranular insula.

Regions of interest (ROIs) were defined based on the Brainnetome atlas (Fan et al., 2016). In that atlas, the cingulate cortex is divided into 7 subregions in each hemisphere. Specifically, the cingulate cortex subregions include the dorsal area 23 (A23d), rostroventral area 24 (A24rv), pregenual area 32 (A32p), ventral area 23 (A23v), caudodorsal area 24 (A24cd), caudal area 23 (A23c) and subgenual area 32 (A32sg). Since prior work has shown that there were no obvious differences in the rsFC of the cingulate cortex subregions in different hemispheres (Yu et al., 2011), bilateral ROIs were formed by combining the cingulate cortex subregions in the left and right hemispheres, resulting in 7 bilateral cingulate ROIs. Similarly, as prior work demonstrated no hemispheric differences in the rsFC of the insula subregions (Cauda et al., 2011), 6 bilateral ROIs in the insula were defined based on the Brainnetome atlas. These insula ROIs include the hypergranular insula (GI), ventral agranular insula (vIa), dorsal agranular insula (dIa), ventral dysgranular and granular insula (vId/vIg), dorsal dysgranular insula (dId), and dorsal granular insula (dIg). Names, voxel sizes and MNI coordinates of the ROIs are listed in Table 1. Figure 2 shows the ROI locations.

**Table 1:** Table of cingulate and insula ROIs.

**Brain region**	**ROI name**	**Size (# of voxels)**	**lh.MNI (X, Y, Z)**	**rh.MNI (X, Y, Z)**
Cingulate	Dorsal area 23 (A23d)	896	−4, −39, −31	4, −37, 32
	Rostroventral area 24 (A24rv)	441	−3, 8, 25	5, 22, 12
	Pregenual area 32 (A32p)	867	−6, 34, 21	5, 28, 27
	Ventral area 23 (A23v)	705	−8, −47, 10	9, −44, 11
	Caudodorsal area 24 (A24cd)	624	−5, 7, 37	4, 6, 38
	Caudal area 23 (A23c)	1,116	−7, −23, 41	6, −20, 40
	Subgenual area 32 (A32sg)	1,010	−4, 39, −2	5, 41, 6
Insula	Hypergranular insula (GI)	611	−36, −20, 10	37, −18, 8
	Ventral agranular insula (vIa)	449	−32, 14, −13	33, 14, −13
	Dorsal agranular insula (dIa)	499	−34, 18, 1	36, 18, 1
	Ventral dysgranular and granular insula (vId/vIg)	561	−38, −4, −9	39, −2, −9
	Dorsal granular insula (dIg)	543	−38, −8, 8	39, −7, 8
	Dorsal dysgranular insula (dId)	623	−38, 5, 5	38, 5, 5

For each ROI, the ROI-based seed signal was calculated by averaging the BOLD signal over the voxels in that ROI. Voxel-wise correlation maps were computed as the Pearson's correlation coefficients between the ROI-based seed signal and the BOLD signal of every voxel in the brain. Correlation values were converted to z-scores using Fisher-z transformation. Two-sided paired t-tests were calculated for the main contrast of interest, (Post-Pre)_*treatment*_ vs. (Post-Pre)_*control*_, based on the z-score maps across subjects. The group *t*-statistic maps were first voxelwise thresholded at *p* < 0.005, and permutation-based multiple comparisons correction was then performed to control the family-wise error rate (FWER) to be less than 0.05 (using AFNI command 3dttest++ with option -ClustSim) (Cox et al., 2017). *Post-hoc* analyses were performed using the mean z-scores of any significant clusters found from the main contrast of (Post-Pre)_*treatment*_ vs. (Post-Pre)_*control*_. To assess the effect sizes, Cohen's *d* was calculated as $d=\frac{t}{\sqrt{N}}$, where *t* is the group t-statistic and *N* is the sample size.

Additional *post-hoc* analyses were performed to verify that the observed rsFC effects between the bilateral ROIs and significant clusters were not driven solely by the ROI signals in the left or right hemisphere. For each bilateral ROI, the Pearson's correlation coefficients between the averaged BOLD signal of the significant cluster and the averaged BOLD signal from an ROI defined using voxels in either the left or right hemisphere were calculated and converted to z-scores using Fisher-z transformation. Then, a two-sided paired t-test was calculated for the main contrast (Post-Pre)_*treatment*_ vs. (Post-Pre)_*control*_ using the z-scores across subjects.

Furthermore, *post-hoc* analyses were performed to examine whether there were any gender differences in the effects found in the seed-based full brain connectivity analysis. Two-sample t-tests were computed for the contrast of male vs. female using the mean z-scores of the significant clusters from the main contrast.

## 3. Results

### 3.1. Participants

Out of 27 total subjects, two dropped out after their first session, due in part to the coronavirus disease 2019 (COVID-19) related suspension of research activities during the course of the study. The remaining twenty-five subjects completed both the control and treatment sessions. Four subjects were excluded from the study due to insufficient volumes after motion censoring (see Section 2.4), leaving twenty-one total subjects included in the analysis. Among these twenty-one subjects, there were 11 females and 10 males, aged 19–40 years (24.6 ± 4.4 years old, mean ± std.). These subjects received a mean stimulation amplitude of 3.28 ± 0.71 mA (min = 1.5 mA, max = 3.8 mA). With eleven subjects having the treatment session first and ten subjects having the control session first, the order of the control and treatment sessions was counterbalanced. The control and treatment sessions were matched to take place at the same time each day whenever possible, with an average time-of-day difference of 0.9 ± 1.25 h (min = 0 h, max = 4 h).

No adverse event or discomfort was reported by the subjects during or after receiving the 20kHz taVNS. No subject reported feeling the stimulation that was delivered at 75% of perceptual threshold. When asked whether they could identify the control and treatment sessions across their two visits, seventeen of the twenty-one subjects responded that they could not guess. Of the remaining four subjects who indicated they could identify their sessions, two guessed incorrectly and two guessed correctly.

### 3.2. Seed-based Full Brain Connectivity Analysis

**Figure 3:**
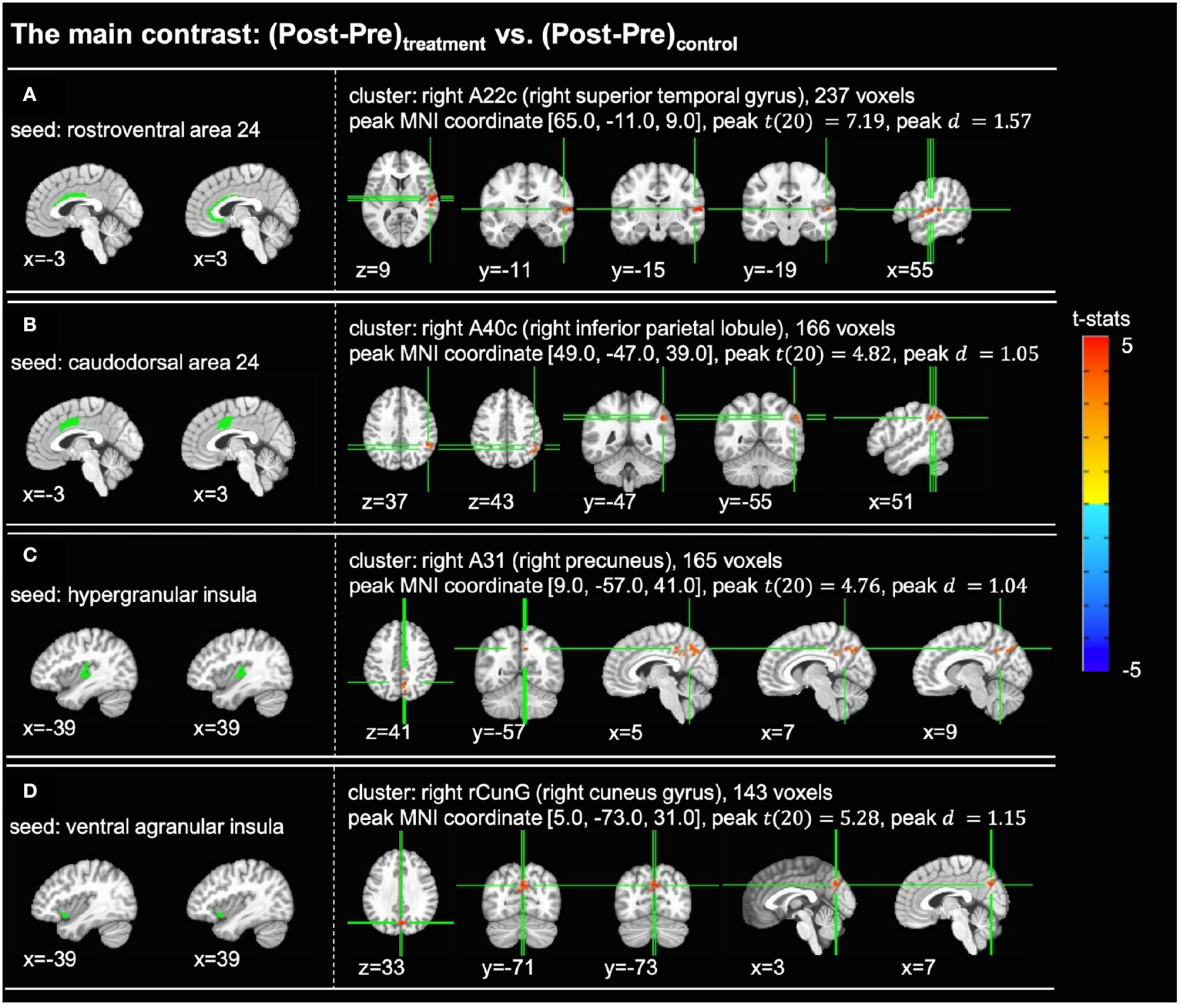
Significant clusters found in the seed-based full brain connectivity analysis for the main contrast of (Post-Pre)_*treatment*_ vs. (Post-Pre)_*control*_ (voxelwise *p* < 0.005 uncorrected, cluster-level *p* < 0.05 FWER corrected). In each subfigure, the left panel shows the location of the seed region and the right panel shows the *t*-statistic map of the significant cluster. **(A)** With the rostroventral area 24 (A24rv) as the seed region, one cluster with 237 voxels was found in the right superior temporal gyrus. **(B)** With the caudodorsal area 24 (A24cd) as the seed region, one cluster with 166 voxels was found in the right inferior parietal lobule. **(C)** With the hypergranular insula (GI) as the seed region, one cluster with 165 voxels was found in the right precuneus. **(D)** With the ventral agranular insula (vIa) as the seed region, one cluster with 143 voxels was found in the right cuneus gyrus. A22c, caudal area 22; A40c, caudal area 40; A31, area 31; rCunG, rostral cuneus gyrus.

For each of the cingulate and insula seed regions, statistical analysis was conducted on the seed-to-voxel functional connectivity maps for the main contrast of interest, (Post-Pre)_*treatment*_ vs. (Post-Pre)_*control*_. For each significant cluster found in the statistical analyses, the location of the peak activation in the Brainnetome atlas was determined by the AFNI command whereami. Figure 3 shows the t-statistic maps and sizes of the significant clusters, as well as the t-statistics, effect sizes, and MNI coordinates of the peak activations. Significant changes in functional connections to the cingulate cortex were detected between rostroventral area 24 (A24rv) and right superior temporal gyrus (rSTG) (Figure 3A) and between caudodorsal area 24 (A24cd) and right inferior parietal lobule (rIPL) (Figure 3B). Significant changes in connections to the insula were found between hypergranular insula (GI) and right precuneus (Figure 3C) and between ventral agranular insula (vIa) and right cuneus gyrus (Figure 3D).

**Figure 4:**
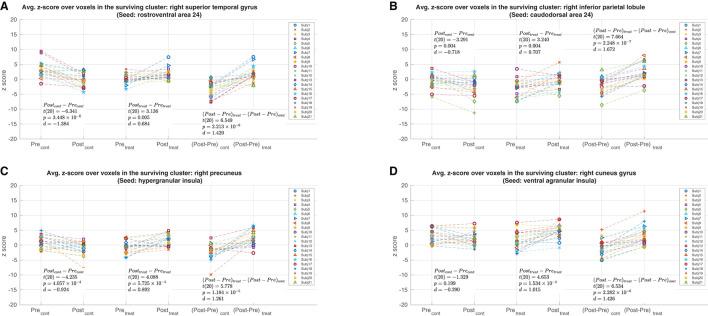
*Post-hoc* analyses with mean per-subject z-score values from the clusters shown in Figure 3. The Pre and Post values from the control and treatment sessions are shown in the leftmost and center pairs, respectively. The (Post-Pre) z-score differences from the control and treatment sessions are shown in the rightmost pair. **(A)** The mean z-score values from the cluster in the right superior temporal gyrus with the rostroventral area 24 as the seed region. **(B)** The mean z-score values from the cluster in the right inferior parietal lobule with the caudodorsal area 24 as the seed region. **(C)** The mean z-score values from the cluster in the right precuneus with the hypergranular insula as the seed region. **(D)** The mean z-score values from the cluster in the right cuneus gyrus with the ventral agranular insula as the seed region.

*Post-hoc* analyses of the mean z-score values for the connections with significant changes are shown in Figure 4. For the clusters found with the A24rv, A24cd, and GI as seed regions, the *post-hoc* analyses (Figures 4A–C) revealed significant increases in z-scores in the treatment session (*p* < 0.01) and significant decreases in z-scores in the control session (*p* < 0.01). For the cluster found with vIa as the seed region (Figure 4D), there was a significant increase in z-scores in the treatment session (*p* < 0.01) alongside a statistically insignificant decrease in the control session (*p* = 0.2). Additionally, *Post-hoc* analyses were performed to determine whether the observed effects were driven by the left or right hemisphere. These tests revealed that the main effects remained significant (*p* < 0.012) for all clusters when using the ROIs confined to one hemisphere (left or right A24rv to rSTG: *p* < 1 × 10^−6^; left or right A24cd to rIPL: *p* < 1 × 10^−6^; left GI to right precuneus: *p* = 0.001; right GI to right precuneus: *p* < 1 × 10^−6^; left vIa to right cuneus gyrus: *p* = 0.011; right vIa to right cuneus gyrus: *p* < 1 × 10^−6^). These results suggest that the main effects were not driven by unilateral effects, and hence support the use of bilateral ROIs in the seed-based full brain connectivity analysis. Furthermore, two-sample t-tests were calculated for the contrast of male vs. female to assess the gender differences in the significant rsFC changes. There were no significant gender differences for the clusters found with the A24rv, A24cd and GI as seed regions (A24rv to rSTG: *p* = 0.497; A24cd to rIPL: *p* = 0.505; GI to right precuneus: *p* = 0.544), while there was a significant gender difference [*t*_(19)_ = 2.873, *p* = 0.0097] for the connection between vIa and right cuneus gyrus.

## 4. Discussion

In the current study, we found that a 15-min treatment of sub-threshold 20 kHz taVNS led to significant increases in resting-state functional connectivity (rsFC) to the cingulate cortex and insula in healthy subjects. These effects were observed using a stimulus paradigm and duration similar to those of the daily dose employed in the recent open label clinical trial of Marsal et al. (2021).

With regard to functional connections to the cingulate cortex, rsFC was significantly altered between caudodorsal area 24 (A24cd) and right inferior parietal lobule (rIPL) and between rostroventral area 24 (A24rv) and right superior temporal gyrus (rSTG). According to the cytoarchitectural based four-region model of the cingulate cortex proposed in Vogt (2005), A24cd is part of the midcingulate cortex (MCC) and A24rv overlaps with the anterior cingulate cortex (ACC) and MCC. Both MCC and ACC have been shown to play a key role in the processing and perception of pain (Derbyshire, 2000; Peyron et al., 2000; Vogt, 2005, 2016; Brown and Jones, 2008; Xiao and Zhang, 2018). The rIPL and rSTG regions that exhibited altered rsFC with the cingulate regions are also thought to be part of the pain neuromatrix (Baciu et al., 1999; Symonds et al., 2006; Kong et al., 2010; Palermo et al., 2014; Budell et al., 2015; Pauw et al., 2019).

Sub-regions within the insula that exhibited altered connectivity were the hypergranular insula (GI) and ventral agranular insula (vIa), which are located in the posterior insula (PI) and anterior insula (AI), respectively. The insula is a key brain region involved in interoception (Craig, 2009) with both posterior and anterior insular regions exhibiting neural responses to pain (Wager et al., 2013). The current study revealed a taVNS-related increase in rsFC between the GI and right precuneus, a pathway that has been observed to have reduced rsFC in migraine patients vs. healthy controls (Yu et al., 2017). Functional connectivity between vIa and right cuneus gyrus was also found to be significantly altered by taVNS. In prior neuroimaging studies, the cuneus has been implicated in the non-visual aversive processing of pain (Fulbright et al., 2001; Matharu et al., 2004; Buhle et al., 2013).

Although the current study was focused on healthy subjects, the findings may have implications for understanding the effects of taVNS in disease states, such as those reported in the clinical trial of RA patients (Marsal et al., 2021). In a rsfMRI study comparing RA patients with healthy controls, Flodin et al. (2016) reported alterations in rsFC involving brain regions associated with pain processing, including the insula, ACC, and MCC. Additionally, a recent study of RA patients showed that higher levels of peripheral inflammation, pain, and fatigue were associated with increased rsFC between multiple brain networks and the left IPL and medial prefrontal cortex (Schrepf et al., 2018). In this study, taVNS induced rsFC changes in brain regions involved in pain processing, including the ACC, MCC and right IPL, were identified in healthy subjects. While taVNS may have different effects on the pain processing regions of healthy subjects vs. RA patients, the current and prior findings provide preliminary support for the potential of sub-threshold high-frequency taVNS to induce alterations in rsFC that are related to improvements in clinical symptoms. Future work is needed to investigate whether similar effects can be observed in clinical populations and to determine whether such effects can be linked to the treatment outcomes of taVNS.

As compared to prior studies examining the effects of taVNS on rsFC, there are a number of key methodological differences in the current study. First, the current study examined sustained rsFC changes in healthy subjects, whereas prior studies have focused on effects in patient populations with major depression, migraine, and insomnia (Liu et al., 2016; Zhang et al., 2019, 2020, 2021; Zhao et al., 2020). Second, we used a sub-threshold high frequency (20 kHz) stimulus whereas prior studies used super-threshold low frequency (1 or 20 Hz) stimuli. There are likely to be frequency dependent neurophysiological mechanisms that lead to differences in the effects between the high and low frequency stimulus paradigms. In addition, the sub-threshold stimulus enabled the use of a sham condition that subjects could not readily differentiate from the treatment condition. Prior studies either did not employ a sham condition (Zhao et al., 2020; Zhang et al., 2021) or used a super-threshold sham stimulus applied to the outer ear (Fang et al., 2016; Liu et al., 2016; Zhang et al., 2019, 2020). Since subjects could feel differences in the stimulus location between the treatment and sham stimuli, the prior studies are subject to somatosensory confounds that are greatly minimized with the approach used in this study. Finally, the current study assessed the effects of 15 min of taVNS, which is on the same order as the 8-min treatment duration used in Zhang et al. (2019) and the 30-min treatment duration used in Zhao et al. (2020). However, the majority of the prior studies (Fang et al., 2016; Liu et al., 2016; Zhang et al., 2020, 2021) have considered the effects of a much longer treatment regime, consisting of 4 weeks of twice-daily 30 min treatments.

Given the significant differences in stimuli and experimental designs, it is not straightforward to compare the current and prior findings. For example, changes observed in prior studies may largely reflect the modulation of functional connectivity in networks most affected by the specific disease of interest. These networks would not necessarily show an effect in healthy subjects. Furthermore, prior findings vary widely due to differences in patient populations and experimental approaches. Nevertheless, it is interesting to review some of the prior findings in the context of the regions examined in this study. In a study of patients with major depressive disorder, Liu et al. (2016) found a taVNS-related decrease in rsFC between the right insula and regions of the default mode network. In a study of migraine patients, Zhang et al. (2019) described taVNS-evoked BOLD fMRI increases in the right anterior insula and decreases in the left posterior insula, however differences in these regions were not significant when compared to sham treatment. In addition, the insula was not identified as a region that showed significant changes in rsFC with the locus coeruleus seed region. Zhang et al. (2021) found that patients with primary insomnia exhibited taVNS-related decreases in rsFC between the dorsal anterior cingulate cortex and the medial prefrontal cortex, while Zhang et al. (2020) reported that migraine patients showed taVNS-related increases in rsFC between the anterior cingulate and a motor-related thalamic subregion. Altered resting-state connectivity to the right precuneus has been mentioned in several studies, including reports of decreased rsFC with the medial prefrontal cortex (Zhang et al., 2021) and with right angular, right superior frontal gyrus, and right middle frontal gyrus (Zhao et al., 2020) in insomnia patients and of decreased rsFC with a vision-related thalamic subregion in migraine patients (Zhang et al., 2020). Taken together, prior studies provide evidence that support the ability of taVNS to alter rsFC in functional networks involving the cingulate cortex and insula. However, further studies are needed to characterize how these effects vary with experimental condition, including disease state and stimulus amplitude, frequency, and duration.

*Post-hoc* analysis of the results (Figure 4) showed that significant effects in the main contrast were consistent with significant treatment-related rsFC increases for all four clusters and significant decreases in rsFC in the control condition for three of the clusters. It is possible that the rsFC changes in the control condition reflect the effects of mental fatigue on the part of the subjects, who were required to maintain a steady level of attention during the eyes-open fixation rsfMRI scans. While the effects of mental fatigue on rsFC are not well-understood, a previous fMRI study reported fatigue-induced deactivation across a broad range of brain regions, including the parietal cortices and precuneus, during visual and auditory tasks (Nakagawa et al., 2013). The significant rsFC increases in the treatment session may therefore reflect a combination of factors, such as a taVNS-related reduction in mental fatigue or an increase in rsFC that is greater than the fatigue-related decrease.

In conclusion, the present results show that 15 min of high-frequency sub-threshold taVNS can lead to sustained changes in rsFC of regions associated with the processing of pain. Future work is needed to determine whether similar effects can be observed in clinical populations, such as patients with RA, and to examine whether such effects can be linked to the treatment outcomes of taVNS. In addition, further work to assess the role of stimulation parameters, such as frequency and duration, would be useful.

## Data Availability Statement

The raw data supporting the conclusions of this article will be made available by the authors, without undue reservation.

## Ethics Statement

The studies involving human participants were reviewed and approved by the UCSD Institutional Review Board (IRB). The patients/participants provided their written informed consent to participate in this study.

## Author Contributions

GH, KM, MF, and TL designed the experiment. YM, CC, and TL wrote and edited the main manuscript text. YM performed the processing and analysis of data and prepared all figures. YM, CC, and MF collected the data used in the study. VS provided hardware support for the study. GH, KM, KA, and TL provided interpretation of the data. All authors reviewed and provided feedback on the manuscript. All authors contributed to the article and approved the submitted version.

## Funding

This work was supported by a research grant from Nēsos Corporation, Redwood City, CA, United States.

## Conflict of Interest

Funding for this study was provided by an unrestricted grant from Nēsos (Redwood City, CA), which also provided hardware support for this study. KM and GH report consultant fees from Nēsos. KA, GH, and VS report equity holdings in Nēsos. KA and VS are current employees. TL reports grants from Nēsos. The remaining authors declare that the research was conducted in the absence of any commercial or financial relationships that could be construed as a potential conflict of interest.

## Publisher's Note

All claims expressed in this article are solely those of the authors and do not necessarily represent those of their affiliated organizations, or those of the publisher, the editors and the reviewers. Any product that may be evaluated in this article, or claim that may be made by its manufacturer, is not guaranteed or endorsed by the publisher.
